# Access to Information Technologies and Consumption of Fruits and Vegetables in South Africa: Evidence from Nationally Representative Data

**DOI:** 10.3390/ijerph17134880

**Published:** 2020-07-07

**Authors:** Sikhulumile Sinyolo, Catherine Ndinda, Conrad Murendo, Sithembile A. Sinyolo, Mudzunga Neluheni

**Affiliations:** 1 Human Sciences Research Council, 134 Pretorius Street, Pretoria 0001, South Africa; CNdinda@hsrc.ac.za (C.N.); MNeluheni@hsrc.ac.za (M.N.); 2 International Crops Research Institute for the Semi-Arid Tropics, Box 776, Bulawayo 263, Zimbabwe; cmurendo@gmail.com; 3 Link Development Analytics, 70079 Zengeza 4, Chitungwiza 263, Zimbabwe; 4 Agriculture Sector Education and Training Authority, 529 Belvedere Street, Arcadia, Pretoria 0002, South Africa; Sithembile@agriseta.co.za

**Keywords:** fruits and vegetables, information access, healthy diets, South Africa

## Abstract

Extensive evidence indicates that fruit and vegetable (F+V) consumption leads to reduced chances of diet related non-communicable diseases (NCDs). However, the F+V consumption levels remain low. This paper investigates the extent to which access to information technologies improves F+V consumption in South Africa. A nationally representative sample of 20,908 households was analysed using the Poisson and logit regression models. The study results indicated that most households do not consume sufficient F+V per day. Only 26% of the household heads consumed F+V at least five times a day. Access to mobile phones, radio, television, and internet was associated with increasing frequency of F+V consumption, and higher chances that a household would consume the minimum recommended levels. The association between the communication technologies and F+V consumption varied. Television access had the highest association with both foods, while internet was only significantly associated with vegetable consumption. Several demographic and socio-economic factors played a key role in shaping F+V consumption patterns. The results show that there is scope to disseminate nutrition awareness and education programs, through mobile phones, internet, radio and television in South Africa. The interventions to promote F+V consumption should be tailored according to the different socio-economic profiles of the population.

## 1. Introduction

Non-communicable diseases (NCDs) are the leading cause of deaths globally and in South Africa [1,2]. According to [1], NCDs accounted for 71% of the 57 million deaths in 2016, of which 15 million of these were premature. The greatest burden of these NCDs is borne by low- and middle-income countries, where 78% of all NCD deaths and 85% of premature deaths occurred in 2016 [1]. This trend is expected to continue, with 80% of NCD related deaths in 2030 expected to be in these poor countries [3]. In South Africa, NCDs contributed to 51% of all deaths in 2016 [1]. Unhealthy diets, which involve a low intake of fruits and vegetables, on one hand, and an excess intake of fat, sugar, and salt on the other, are a major cause of diet related NCDs in many countries, including in South Africa [2,4,5,6,7,8,9]. According to [7], the risk posed by unhealthy diets to morbidity and mortality exceeds that of the combined effect of unsafe sex, and alcohol, drug, and tobacco use. 

Extensive evidence [7,10,11] shows that increased intake of the fruits and vegetables is associated with reduced chances of diet related NCDs (i.e., cancer, diabetes and cardiovascular diseases) and mortality. Accordingly, the WHO has been on the forefront of promoting increased fruit and vegetables for healthy living. However, the consumption of fruits and vegetables remain low globally, 20 to 50 per cent below the minimum 400 g, or five 80-g portions, a day, as per WHO’s recommendations [6,12,13]. A recent study conducted in 49 low-and-middle-income countries, for example, found that less than 30% of adolescents across all the 49 countries met the WHO guidelines for fruit and vegetable (F+V) consumption [13]. Similar results have been reported in South Africa, where various studies [14,15,16] have found that South Africans consume fruits and vegetables less frequently than the minimum daily recommended level of five times a day, and smaller portions per serving than the target 80 g. For example, [15] found that, on average, their study participants consumed less than 200 g of F+V per day, which is 50% short of the minimum recommended level. The [14] reported that only 38% of their study participants consumed at least two portions of F+V daily. The 2018 General Household Survey [17], which is analysed in our study, indicated that 26% of the household heads consumed F+V at least five times a day. Limited availability, accessibility and affordability of F+V partially contribute to the inadequate intake of fruits and vegetables [18,19]. Another key reason for the low fruit and vegetable consumption is limited awareness and knowledge on the health benefits of fruit and vegetables [12,19,20]. 

Finding strategies to improve the consumption of nutritious diets, particularly diets rich in F+V, is firmly on the policy agenda in South Africa. This is highlighted in various policies, strategies and programmes (e.g., Integrated Nutrition Programme, National Food and Nutrition Security Policy, South African Guidelines for Healthy Eating and the Food Guide, the 2015–2020 Strategy for the Prevention and Control of Obesity in South Africa, the 2020–2025 National Strategic Plan for the Prevention and Control of Non-Communicable Diseases, etc.) [21,22] Among other interventions, such as increasing the availability and affordability of F+V, these strategic documents emphasise the importance of raising awareness and educating the public on nutritious diets and healthy lifestyles. Assumed in these strategies is that limited nutrition awareness or knowledge is a key barrier to dietary improvement, such that improving access to nutrition information should result in better diets. Household visits and facilitating community dialogues are two approaches adopted by the South African government to increase health and nutrition awareness and promote healthy behaviours. While these approaches are effective, they do not reach many people and are too expensive to scale up in resource-limited settings [8,23,24,25]. 

The interventions outlined in South Africa’s health and nutrition strategic documents therefore include the use of mass media communication technologies, such as radio, television, online and print media to improve nutrition education and awareness, with a view of reaching large audiences. In recent years, there has also been a focus on the use of social media and web-based applications [26]. This is in line with [27], who encouraged policy makers worldwide to use mass media channels in implementing policies and strategies which aim to promote the consumption of nutritious foods. While the use of mass media campaigns has been implemented across various programmes as a key strategy to improve the public’s health and nutrition knowledge, it is not clear whether this approach has achieved much success. The reach and success of the health and nutrition massages delivered through mass media depend on the extent to which the consumers have access to the communication technologies to receive the information. For example, information delivered through television has a chance of raising nutrition awareness of those that own, or have access to, a working television set. Similarly, individuals with access to the internet are the ones mostly likely to access information shared via social media and websites of relevant institutions or other electronic media. The huge number of households with access to information and communication technologies, such as mobile phones, radio or television internet, suggests that the information shared through these channels is potentially reaching many in the country [17]. 

The extent to which information accessed through these different channels has resulted in improved diets has not been adequately investigated in South Africa. While studies have looked at the determinants of F+V consumption in South Africa [14,15,16,28] and in other developing countries [29,30,31,32], these have generally not investigated the effect of information access. There is growing literature [33,34,35] that links access to information through mobile phones to nutrition. These studies have mainly focused on dietary diversity score as a nutrition indicator, and not specifically the consumption of fruits and vegetables. Dietary diversity scores are not necessarily a good indicator of diet quality [33,36]. It is possible, for instance, for an individual to achieve a high dietary diversity score, and yet their diet to be deficient in F+V. As [36] argued, it is not necessarily the number of food groups that matters for healthy nutrition, but the supply of all essential nutrients in sufficient quantities. As such, it is important that literature on nutrition go beyond dietary diversity and investigate the other dietary indicators, such as the consumption of F+V, which are important sources of micronutrients [36]. In addition, factors that might be positively associated with dietary diversity might not be significantly correlated with specific food groups such as F+V [36,37,38]. For example, whereas [38] found significant and positive correlations between farm production diversity (as measured by the Simpson’s index) and dietary diversity scores, F+V consumption was not significantly associated with farm production diversity. 

Further, the nascent literature linking information technologies and nutrition [33,34,35] has mainly focused on one information technology (particularly mobile phones), and has not compared the effectiveness of the different communication modes in improving healthy diets. Studies that have investigated the effects of different communication modes (text, audio or video) in modifying health behaviours [18,39,40,41,42,43] found mixed results. For example, a systematic review by [42] reported that video interventions were more effective in modifying behaviours related to the learning of new behaviours (e.g., breast self-examination, prostate cancer screening, etc.), than in stopping harmful ones (e.g., addiction behaviours). Findings by [43] showed that videos were more effective than text in encouraging people to quit smoking, while [40] found that health communication using testimonials was more persuasive when presented through the audio mode than when presented in written mode. A study by [41] found that a mobile app that used audio resulted in a higher level of F+V consumption than one that relied on textual messaging. While these studies have mostly relied on experiments, policy makers need to know which communication channel works better in practice for the promotion of a specific food type, to inform their choices of specific mass media mode. 

This study uses survey data to investigate the relationship between four communication technologies (mobile phones, radios, television and internet) and the consumption of F+V in South Africa. While most of the studies investigating the F+V consumption in South Africa have relied on small and unrepresentative samples, we use the 2018 General Household Survey [17], a nationally representative sample of 20,908 households collected by the national statistics agency, Statistics South Africa (Stats SA). The study compares the effect of the different communication technologies on the consumption frequency of F+V. Our study seeks to build on literature that has investigated the determinants of fruits and vegetables, by focusing on the role of information technologies, which have often been neglected by these studies. We also seek to build on the literature that has focused on the role of mobile money on nutrition, by expanding the number of communication technologies beyond mobile phones and focusing specifically on F+V consumption.

## 2. Materials and Methods

### 2.1. Conceptual Framework

We identify two main pathways through which communication technologies such as mobile phones, television, radio and internet positively link to the consumption of F+V. Firstly, households who own, or have access to, communication technologies are expected to have easier access to nutrition information. Access to information is expected to increase nutrition knowledge and awareness, leading to behavioural changes and improved dietary practices. Literature [33,44,45] has reported that access to relevant nutrition information is associated with increased purchases and consumption of healthier foods. Some studies that have investigated the effectiveness of mass media campaigns on reducing health-risk behaviours have reported changes in behaviour. For example, a review by [46] found that mass media campaigns produced positive changes in health-related behaviours (such as reducing smoking, drugs, etc.) across large populations. In South Africa, [47] found that a mass media campaign to increase the awareness of the importance of reducing salt intake led to a shift in attitudes and behaviours, and a reduction in salt consumption. Given the increased sharing of information on the benefits of consuming F+V, particularly its linkages to NCD prevention, it is expected that those with access this information will be aware and motivated to consume these foods more frequently than those with no or limited information access. 

Secondly, the ownership of communication technologies improves coordination and reduces information search and other transaction costs [34]. This includes reducing transaction costs related to the purchase of F+V, as well as other household activities. For example, access to the Internet, or a mobile phone, allows a household to search easily for information that relates to the availability, location and prices of F+V, or any other commodity the household is interested in, instead of incurring transport costs driving to the market, only to find that the commodity is not available. The reduction in costs incurred by households leads to increased net savings, which they can spend on F+V. For households that produce F+V, the ownership of communication technologies improves access to information on the input and output markets, as well as technologies that could lead to increased productivity [34]. Though access to nutrition information on the health benefits of F+V is important, it is not sufficient on its own to lead to high levels of F+V consumption. Other factors, such as incomes, education, gender, etc., also play significant roles in shaping the willingness and ability of households to consume F+V. The models estimated in this study control for some of these factors.

### 2.2. Data 

This study used a mixed method, adopting both quantitative and qualitative approaches. The quantitative data analysed is from the 2018 South Africa General Household Survey [17], collected by Statistics South Africa (Stats SA), the national statistics agency. The survey targeted all private households in all nine provinces in South Africa, with the exception of those households living in collective living quarters, such as hospitals, prisons or military barracks. The details of the sampling approach are presented in [17], and we present a brief summary. The survey employed a complex, stratified two-stage sampling design. Primary stratification was done using variables such as province, geographical area (metropolitan and non-metropolitan), gender, education, income, etc. Primary sampling units were selected within strata, using probability proportional to size. The measure of size was the number of households. A total of 3080 primary sampling units were selected. 

In each selected primary sampling unit, a systematic sample of dwelling units was drawn. Dwelling units refer to any structure or part of a structure or group of structures occupied, or intended for occupation, by one household [17]. A varying number of dwelling units was selected per primary sampling unit. The final sample consists of 20,908 households. The sampling weights for the data were constructed so that the sample is representative of the non-institutionalised and non-military households in South Africa. The data were analysed using Stata 15. The analysis in this study took into account the sampling weights, and the results are therefore representative of all household heads in South Africa. However, according to [17], caution should be exercised when the results of the survey are interpreted at low levels of disaggregation. This study does not attempt to interpret results below the national level.

To give contextual meaning to the quantitative results, and to understand diverse perspectives on the drivers and inhibitors of F+V consumption in different settings, focus group discussions (FGDs) and key informant interviews (KIIs) were done at selected sites in Gauteng, Mpumalanga, and KwaZulu-Natal provinces. The three provinces were purposively selected, with Gauteng selected because it is highly urbanized; Mpumalanga selected because it is semi-urbanized, while KwaZulu-Natal was selected because it is one of the less urbanized/more rural provinces in the country. Three study sites were selected per province to represent urban, semi-urban and rural settings, resulting in nine research sites across the three provinces. Officials who work in government departments (such as departments of agriculture and rural development, health, economic development, trade and industry), private health practitioners, officials from, and members of, the South Africa NCD Alliance, community leaders and community-based organisation representatives were selected as key informants. The key informants were selected based on their knowledge and understanding of the consumption of fruit and vegetables at the local level, and involvement in the implementation of programmes and the design of policies that influence the consumption of fruit and vegetables in the selected regions. Twenty-three (23) key informants were interviewed in total across the sites. Local actors, particularly community leaders and community-based organization assisted in organizing participants in focus group discussions. The criteria was to select participants with different demographic and socio-economic profiles (age, gender, education, employment status, etc.) to ensure a greater understanding of the norms, values, experiences and changes taking place in the communities with regard to the consumption of fruit and vegetables. Eighteen (18) focus group discussions were conducted across the three provinces, involving between 6–10 participants. In total, 132 participants took part in the focus group discussions. We do not present separate results from the FGDs and KIIs, but we use insights from these to explain the statistical results.

### 2.3. Measurement of Key Variables

The dependent variables were captured in terms of how frequently the household heads consumed fruits or vegetables. The variables were generated from questions that asked the respondent to indicate how often they had consumed different food types over a 24-h recall period. The vegetables include spinach, cabbage, carrots, relish, tomatoes, beetroot, beans, peas, groundnuts, cashew nuts, etc., while fruits include oranges, mangoes, guavas, etc. The potatoes were excluded, as per WHO recommendations. The dependent variables were also captured in a binary format, showing whether a household head consumed the WHO recommended minimum levels of fruit (at least two servings) and vegetables (at least three servings), or both (at least five servings). It should be noted that the dependent variables used in this study have some limitation. The food consumption frequencies, despite their popularity in the literature [48], are not a very precise indicator of the amount of F+V consumed. The size of the servings was not captured in the survey, and there is no guarantee that each serving was equal to 80 g [36]. As such, the reported frequencies in this study might not be equal to the WHO recommended 400 g per day, even when a respondent meets the five times a day rule. The consumption frequencies in this study only concern household heads and are not necessarily representative of the consumption levels of households. The food consumption questions were only asked to household heads in the survey. As such, the available data does not permit us to study the consumption patterns of other household members or intra-household dynamics.

Four variables were used to proxy access to information technologies, which were ownership of mobile phones, internet, radio and television. The survey asked respondents to indicate if their households own these communication technologies, among other household assets. The other explanatory variables were selected based on literature and the availability of data, and included the household head’s demographics (e.g., age, gender, education and race) and the household’s socio-economic characteristics (household size, number of females aged 15 years and above, children below 5 years, income levels and main sources, livestock size, etc.) Literature [29,49] has reported that the composition of a household influences foods consumed, with households with an increasing number of females or children under five years expected to consume more F+V than their counterparts. The education level of other household members was considered, since other members can also influence household diets. We therefore included in the model both the household head’s highest education level, and the proportion of household members who completed at least matric (Grade 12). In preparing the education level variable, individuals who did not attend school at all or did not complete grade 1 were scored a zero, while others were scored according to their highest grade or NQF level qualification completed. For example, those who completed grade 5 received a score of 5, while those who completed matric (grade 12 or NQF level 4) were scored 12. The highest level of education (PhD qualification—NQF 10) scored 21.

Income was captured in terms of total household income per month. The main sources of household income were also included, as literature has shown that incomes from different sources are spent varyingly across household needs [50,51]. The household’s wealth status was also captured. The households were asked to rank themselves in terms of their wealth status, and they were categorized into three wealth levels of wealthy, medium and poor. Livestock, captured in tropical livestock units, was also used as a proxy for wealth, as well as a source of animal protein. Vehicle ownership was considered as proxy mobility and ease of transportation. Given that F+V are highly perishable, a household’s ownership of a refrigerator was considered important [52]. A household’s own production of F+V was included in the models, with the assumption that those who produce F+V for themselves would consume more, due to their availability [34]. Literature has shown that urban and rural households’ consumption patterns are different. Distance to a medical facility was considered important, as households located near these facilities have better access to health information than those located faraway.

### 2.4. Data Analysis

#### 2.4.1. Poisson Regression Model

A Poisson model was estimated when the consumption frequencies/counts were used as dependent variables. Two Poisson models were estimated, using the frequency of fruit or vegetable consumption as dependent variables, respectively. The limitation of the Poisson model is that it imposes a restrictive assumption that the conditional variance equals the conditional mean. Observed data often violates this equidispersion assumption, usually displaying pronounced overdispersion (i.e., the variance greater than the mean). However, the mean and variance were not very different in this study (Table 1), indicating little evidence of overdispersion. The Poisson model was therefore preferred over the negative binomial model, as the former makes fewer assumptions than the latter [53].

**Figure 1:**
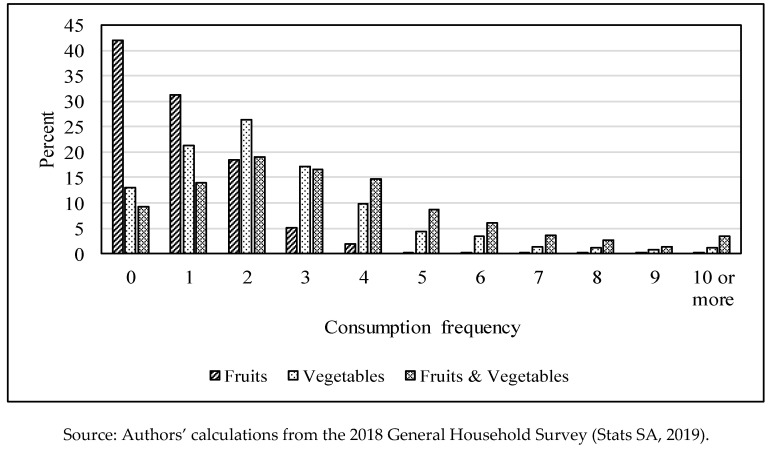
Frequency of fruits and vegetable consumption.

A standard Poisson model was estimated instead of a zero inflated Poisson model, because there was not much preponderance of zeros in the data. Figure 1 shows that less than 10% of the respondents did not consume any fruit or vegetable over the 24-h period, while about 13% had not consumed any vegetables. While there is some evidence of many zeros for fruits consumption frequency, the results from the zero inflated zero Poisson model did not differ much from that of the standard model. A standard Poisson model was estimated for both of the two dependent variables.

The latent Poisson model was estimated as follows:

(1) $$y_{i}^{*}|x\sim P(y_{i}^{*}|x),$$

(2) $$E\left(y_{i}^{*}|x\right)=\exp\left(\alpha +x_{i}^{\prime }\beta \right)=\lambda_{i},$$

where: $y_{i}^{*}$ is the latent fruit or vegetable consumption frequency variable, $x_{i}$ is a vector of information access, demographics and socio-economic characteristics, α and β are coefficients and $\lambda_{i}$ is the mean.

#### 2.4.2. Logit Regression Model

The logit model was used to model the probability that a respondent would meet the minimum recommended daily consumption levels of fruits (at least 2 servings), or vegetables (at least 3 servings). A logit model was estimated for each of the two dependent variables, resulting in two estimations. The logit model was implemented as follows [53]:

(3) $$d_{i}^{*}=w_{i}^{\prime }\delta +u_{i},$$

(4) $$d_{i}=1\left(d_{i}^{*}>0\right),$$

(5) $$\mathrm{Prob}\left(d_{i}=0{|w}_{i}\right)=\pi_{0}\left(w_{i}^{\prime }\delta \right),$$

(6) $$\mathrm{Prob}\left(d_{i}=1{|w}_{i}\right)=1-\pi_{0}\left(w_{i}^{\prime }\delta \right),$$

where: $d_{i}^{*}$ is the latent selection variable which equals 1 when an individual consumed the minimum WHO recommended levels of fruits, or vegetables, 0 otherwise; $w_{i}$ is a vector of covariates, δ are coefficients to be estimated, π is the cumulative probability distribution and $u_{i}$ are the residuals. The dependent variable for the first estimation took the value of 1 if a respondent consumed fruits at least twice, and 0 otherwise. The second dependent variable took 1 if a respondent consumed vegetables at least 3 times; 0 otherwise. Potential endogeneity issues were not controlled for in both the logit and Poisson regression models. As such, the estimates should not be interpreted as causal, but as correlations. 

## 3. Results and Discussions

### 3.1. Descriptive Results

Table 2 presents the means/proportions of the consumption and information access variables. Table 2, and Figure 1, show that, on average, the daily consumption frequencies of fruits, vegetables or both, were below the recommended minimum levels. The table indicates that respondents consumed fruits about once in the period under review, lower than the recommended twice a day. Less than a third of South African household heads consumed fruits at least twice a day, suggesting that fruits are not consumed as regularly as they should be in South Africa. Figure 1 indicates that fruits are rarely consumed more than 4 times among households. The situation is relatively better for vegetables, as only 40% of the household heads consumed them, achieving an average of 2.5 times a day. However, this is below the recommended servings of three times a day, suggesting that over two thirds of household heads in South Africa do not consume vegetables at least three times a day. Overall, the household heads consumed lower than the five a day target, with only 26% of them achieving the recommended minimum. Participants in the FGDs and KIIs identified a number of reasons why there was a limited consumption of F+V. Among other reasons (such as F+V availability, affordability, taste, etc.), the lack of information or knowledge on the health benefits of F+V was one of main reasons explaining limited F+V consumption. For some, the consumption of vegetables, particularly the traditional leafy vegetables, was a sign that one is too poor to afford meat, for example. Another reason that was presented for the limited F+V consumption was because of the fast food culture. According to participants that were interviewed, most families, particularly those where both the husband and wife are employed, rarely cook, and they frequently consume fast foods. Unfortunately, most of the fast food rarely includes health foods such as vegetables.

Table 2 indicates that mobile phones are widely owned in South Africa, with over 96% of households owning at least one mobile phone. On average, a household owned over two mobile phones, with one household reporting ownership of 45 mobile phones. Over two thirds (62.1%) of the households reported having access to the internet, mainly through mobile phones. A further analysis indicated that 60% of households accessed the internet through their phones. There were very few households (10%) who reported that they had broadband internet connections in their houses, while 22% accessed the internet at workplaces, universities, and public WiFi or internet cafes. Participants in the FGDs and KIIs also indicated that most of them accessed the internet through mobile phones. However, it was also indicated that access to the internet through mobile phones was mainly for social media activities, and rarely do people access government websites, except for job search activities among the younger generation. The majority of households (88%) owned at least one television set, while just above half owned a radio. The discussions with participants in FGDs indicated that, while they have seen one or two advertisements on television, the dominant advertisements are for less healthy foods (e.g., potato chips, burgers, etc.)

Table 3 presents a summary of the socio-economic characteristics of South African households. The results show that mostly middle-aged men, the majority (81%) of which belonged to the African race, headed the South African households. On average, the highest education level attained by the household heads was grade 10. The households were small, on average comprising just over three household members. There was at least one female member aged 15 or above in every household. A proportion of 35% of household members had completed at least matric. Households reported modest income earnings per month, on average, which varied from zero for some households to R40,000 for others. A significant share of households (44%) were beneficiaries of social grants, and 20% of households reported that social grants were their main income source. Eighty-eight percent of the household heads were employed, and salaries were the main income source for 59% of the households. About 15% of households were involved in farming activities, and farming was the main income source for 4% of the households. Some farming households were involved in the production of F+V, comprising 7.9% of all households in South Africa. The households reared few animals on average. Most of the households (70%) resided in urban areas in South Africa, suggesting that the country is becoming highly urbanised. Just over 30% of households were in possession of a motor vehicle. Most of the households (43%) reported that they stayed less than 15 min away from a medical facility. A few households (15%) were located at least 30 min away from a medical centre.

### 3.2. Empirical Results

Table 4 presents the factors associated with the frequency of consumption of F+V, and the chances that a household head would consume vegetables at least three times a day or fruits twice a day (the minimum WHO recommended levels). The information technologies and socio-economic factors in the table were mutually controlled for in estimated models, and the estimated coefficients of individual variables satisfy the *ceteris paribus* (holding other factors constant) condition. The results show that the four variables capturing access to information were highly significant and positive across the models, with the exception of internet, which was not significant in the fruits models. The positive and significant estimated coefficients of the information variables indicate that access to information is strongly associated with the increased frequency of F+V consumption, and the probability of consuming the minimum WHO recommended levels of F+V. These findings are consistent with the literature [8,54,55], which has reported that ownership of these communication technologies facilitates easy access to information on the health benefits of the consumption of F+V. Increased awareness of the health benefits of these foodstuffs is more likely to motivate households to consume them more than in the absence of awareness. Access to information also reduces transaction costs, particularly search costs, and improves access to the market, resulting in households with more disposable income to purchase foods such as fruits and vegetables [34]. Given that F+V are expensive, it is important that households get information about where to source these at more affordable prices. In addition, access to information also helps households to be aware of the areas that sell better quality and tastier fruits and vegetables. The quality of fruits and vegetables is an important determinant of their consumption.

In line with the previous literature [18,39,40,41], the results show variations in the associations between the different communication modes and the incidence and probability of consuming F+V. This indicates that different communication modes have a heterogeneous influence on the persuasiveness of health and nutrition messages [18]. The association of television was higher than that of all the information access variables. This is in line with several studies in the literature [42,43], who found that video messaging is more effective than audio or text. The results show that the association of television was stronger for fruits consumption than for vegetables consumption (in both statistical significance and magnitude). This result suggests that, while televisions can be used as a medium for promoting both F+V consumptions, they are more effective in promoting fruits consumption. The shape and colour of a fruit are important in attracting consumers, and less so for most vegetables. Studies such as [56] show that food colour play an important role in acceptance or liking of food items such as fruits, and hence, ultimately, food intake. The role of mobile phones and radio was largely consistent across the models, suggesting that these two communications can be used equally to drive the consumption of both F+V. Access to internet was strongly associated with increased incidence and probability of vegetable consumption, achieving the highest magnitude on the probability (6.1%) of meeting the WHO minimum vegetable consumption level. However, it was not significantly correlated with the frequency of fruits consumption and the probability of consuming the minimum twice a day, as per the WHO’s recommendations. This result suggests that the Internet could be effective in the promotion of vegetable consumption, but not of fruit. 

Furthermore, the results in Table 4 show the largely varied associations of different demographics and socio-economic variables on the frequency of F+V consumption. The positive estimated coefficient of age indicates that older people were more likely to consume diets that included vegetables than younger people are. This suggests that as people become older, they become more conscious about their diets, due to the likelihood of other health challenges related to old age. However, this only applies to vegetable consumption, and not fruit consumption. Interestingly, an increasing number of children under the age of 5 was associated with increased chances of households consuming fruits, and not vegetables. This is unsurprising, since young children prefer fruits (most of which are sweet) than vegetables, such that households with children are likely to buy more fruits than those without children. The increased availability of fruits in the household improves the chances that even adults might end up eating them as snacks. Table 4 shows that the F+V consumption patterns of South Africans differed according to race. The results show that people of colour had a 4% and 16.5% chance of meeting the daily WHO targets of F+V consumption, respectively. People of colour consumed F+V less frequently than Africans, and had significantly less chances of meeting the minimum WHO recommended levels than all other races. In contrast, White people were more likely to consume more vegetables than the other three races, suggesting high nutrition awareness and knowledge among White South Africans. A study by [57] found that White people had significantly more general nutrition knowledge than Black people. A previous study by [16] also reported the insufficient intake of fruits and vegetables among African people or people of colour compared to their Indian and White counterparts.

Unsurprisingly, bigger households were associated with decreasing frequency and chances of meeting the daily consumption targets for F+V. Holding other factors constant, bigger households require more resources to cater for the needs of all household members than smaller households. As such, members of bigger households are less likely to consume adequate F+V compared to members of smaller households. While several studies [49,58,59,60,61] indicated that females are more inclined towards healthier diets than males, Table 4 suggests that this is not the case in South Africa, especially concerning vegetable consumption. The insignificant estimated coefficient of gender on the vegetable consumption models indicates that there were no significant differences in both the frequency of vegetables, and the likelihood of consuming the minimum recommended level, between female and male household heads. However, even though weakly significant, there is evidence that the male household heads consumed fruits less frequently than their female counterparts did. An interesting result in Table 4 suggests that an increased number of females aged 15 years or older in a household was associated with improved chances of a respondent consuming vegetables at least three times a day. Additionally, the number of female household members was associated with the increased frequency of fruit consumption. This result suggests that the gender of the household head matters little when it comes to household dietary decisions. Instead, the presence of female members matters. Females are the ones who make most of the decisions when it comes to what a household purchases, prepares and consumes, even in cases where they are not household heads.

Whereas the education level of the respondent did not make a significant difference in the consumption levels of vegetables, it was positively correlated with fruit consumption. A similar result in South Africa was reported by [58]. This result was not expected, as the expectation was that education level would be associated with the consumption of both F+V, as has been reported by several studies [61,62,63,64]. However, households with a higher proportion of its members who completed matric consumed more fruits and vegetables than those with a lower proportion, which suggests that the education level of other household members is important in influencing diets. Education proxies’ nutrition knowledge as well as the ease of understanding health information, and the increased education levels of household members, lead to the increased access to information, as well as an increased understanding of such information.

In line with expectations and the literature [29,61,63,65], increased incomes were positively correlated with the increased frequency of both F+V consumption. The association was largely consistent for both F+V. However, the major source of income had a bearing on the frequency of F+V consumption. While remittances and farming as major income sources had no significant role, Table 4 shows that households whose main income was social grants consumed both F+V more frequently than households reliant on salaries did. In contrast, households that depended on businesses as the major income source were less likely to consume both F+V. Whereas there was no significant difference in the vegetable consumption frequencies of both wealthy and not-wealthy-not-poor households, the poor households had a 2.6% higher chance of consuming vegetables three times a day than the not-wealthy-not-poor households. On the other hand, the wealthy households consumed fruits more frequently than the not-wealthy-not-poor households, while the poor households consumed less fruits. This suggests that, in line with views shared by participants in the FGDs, vegetables are the poor man’s food, while fruits are for the rich. Increasing livestock size, another proxy for wealth for particularly rural households, was associated with increasing frequency of vegetable consumption, and increased chances of meeting the daily target of fruits consumption. A plausible explanation is that fruits are among the key food items contributing to high overall costs of healthy diets and hence their consumption is most likely to be reduced by poorer households. In addition, poor households use vegetables as relish, while fruits are considered as a snack. Studies such as [66] and [65] also found that poorer households are less likely to consume fruits than richer households. 

In line with expectations, households who own a motor vehicle consumed both F+V more frequently than those who do not own a motor vehicle did. This is because ownership of a vehicle reduces transport costs and improves mobility, which leads to enhanced chances of these households accessing cheaper or better-quality fruits and vegetables. Additionally, given the perishable nature of the F+V, access to reliable transportation increases the chances that a household will be able to arrive to their homes with the F+V still fresh. The results also show that, given the short life span of F+V, households with access to a refrigerator consumed both F+V more frequently than those without a refrigerator did. Studies such as [52] have shown that having access to a refrigerator is associated with higher F+V consumption. A lack of a refrigerator is more likely to result in increased food waste and reduced frequency of consumption of these perishable foods. Discussions with participants in FGDs indicated that they preferred buying non-perishable foods, which are easier to store, than buying perishable foods such as F+V.

The study’s results show that households who produce F+V are more likely to consume vegetables more frequently than those who do not, and had a 6.7% chance of meeting the daily target for vegetable consumption. This is expected, as their own production of vegetables improves their availability and access. Given that affordability of F+V was identified during our FGD and KII discussions, and has been cited by several studies [12,20,58,67] as one of the key factors that constrain consumption in healthy diets, own production ensures that these households do not face the cost barrier. However, the results suggest no significant association between own production and fruit consumption. On one hand, this result suggests that own production only leads to increased own consumption for vegetables, and that the production of fruits might be mainly targeted at the market. On the other hand, it could be because most of the households did not produce fruits, but vegetables. Unfortunately, we are unable to separate the individual associations of F+V consumption, given that the survey did not ask households to state separately which, between the two, they produced.

Urban households consumed vegetables less frequently than rural households. As indicated previously, discussions during the FGDs and KIIs indicated that the contemporary urban lifestyle was such that urban dwellers prefer quick and instant meals; the preparation of vegetables, for instance, was considered an unnecessary inconvenience for the urban working class. This can also be linked to the fact that households in urban areas usually have no gardens and enough space to grow own vegetables and rely on supermarket purchases, which depend on availability and affordability. Households that stay closer to medical facilities consumed F+V more frequently than households that stayed further from these centres. Health institutions disseminate information on nutrition education to patients and pregnant women and this could explain these results. Maternal and child nutrition education from health institutions emphasise the consumption of micronutrients found in F+V. For example, [68] mentioned that the consumption of F+V is promoted as part of a healthful diet, given that maternal nutrition is important for foetal growth. The evidence supports the maternal consumption of a variety of F+V as part of a balanced diet throughout pregnancy [68,69]. Nutrition education also enhances F+V consumption, as alluded by [70]. In addition, many clinics grow vegetables in the community, meaning that people can access free vegetables.

## 4. Conclusions

A sufficient intake of fruit and vegetables has been associated with reduced risks of adverse health conditions. However, the consumption of F+V remains low in South Africa, as in many countries. As such, the search for strategies to improve the consumption F+V is firmly on the policy agenda in South Africa. The interventions outlined in South Africa’s health and nutrition strategic documents include the desire to use mass media communication technologies such as radio, television, online and print media to improve nutrition education and awareness, with a view of reaching large audiences. This study has investigated the association between information technologies (mobile phone, radio, internet and TV) and F+V consumption using survey data. In the present study, it was found that the majority of South African households consumed inadequate amounts of fruits and vegetables. The results show that access to mobile phones, radio, television, and internet was associated with an increasing frequency of F+V consumption, and higher chances that a household would consume the minimum recommended levels. However, the association of the communication technologies and F+V consumption varied. Television access had the highest association on both F+V consumption, while Internet was only significantly associated with vegetable consumption. The study findings suggest that, while sharing nutrition information using most of these communication technologies is likely to improve F+V consumption, the magnitude of the association depends on the specific communication technology used. The study also showed that demographics and socio-economic factors play an important role in shaping the F+V consumption patterns of the people. For example, being Black or a person of colour, bigger households and residence in urban areas were associated with inadequate F+V intake. Income, wealth status, and vehicle ownership were significantly associated with adequate fruit and vegetable consumption. Interventions that enhance income growth should be promoted, as these are positively associated with fruit and vegetable consumption. This study indicates the need for using pluralistic information dissemination channels for promoting nutrition awareness and knowledge and healthy eating practices in South Africa. There is scope to disseminate nutrition awareness and education programs through mobile phones, internet, radio and television in the country. Own production is associated with improved fruit and vegetable consumption. Therefore, programs that promote nutrition gardens and orchards, both in rural and urban areas, should be strengthened. The study findings suggest that interventions to promote F+V consumption should be tailored according to different socio-economic profiles of the population. A one-size-fits-all approach is less likely to succeed in South Africa.

## Figures and Tables

**Table 1:** Mean and variance of the fruit and vegetable consumption variables.

Variable	Mean	Variance
Freq of fruit consumption	0.951	1.167
Freq of vegetable consumption	2.440	2.110
Freq of fruit and vegetable (F+V) consumption	3.386	2.779

Source: Authors’ calculations from the 2018 General Household Survey (Stats SA, 2019).

**Table 2:** Summary statistics of consumption and information access variables.

Variable	Mean	Min	Max
Dependent variables			
Freq of vegetable consumption	2.481	0	28
Prop who consumed vegetables 3 times per day	0.393	0	1
Freq of fruit consumption	0.991	0	10
Prop who consumed fruits twice per day	0.272	0	1
Freq of F+V consumption	3.464	0	35
Prop who consumed F+V 5 times a day	0.261	0	1
Information technology variables			
Mobile_fon	0.966	0	1
Mobile_fon_no	2.351	0	45
Internet	0.621	0	1
Radio	0.526	0	1
Television	0.882	0	1

Source: Authors’ calculations from the 2018 General Household Survey (Stats SA, 2019).

**Table 3:** Sample demographics and socio-economic characteristics.

Variable	Description	Mean	Min	Max
Age	Age of household (HH) head	45.72	12	108
Gender	Gender of HH (1 = Male, 0 = Female)	0.584	0	1
African	Race of HH head_1 (1 = African, 0 = Otherwise)	0.809	0	1
People of colour	Race of HH head_2 (1 = People of colour, 0 = Otherwise)	0.071	0	1
Indian	Race of HH head_3 (1 = Indian/Asian, 0 = Otherwise)	0.024	0	1
White	Race of HH head_4 (1 = White, 0 = Otherwise)	0.095	0	1
HH_size	Household size	3.235	1	22
Females_15	Number of females 15 years or older in HH	1.210	0	11
Children_5	Number of children 5 years and below in HH	0.372	0	6
Educat	Education level of HH head (Years)	10.25	0	21
Matrics_prop	Prop of HH members who matriculated (completed grade 12)	0.354	0	1
Employed	Employment status of HH head (1 = Employed, 0 = Unemployed)	0.880	0	1
Social grants	At least one HH member receives a social grant (1=Yes, 0=No)	0.443	0	1
Farmer	Household engaged in farming activities (1 = Yes, 0 = No)	0.148	0	1
F+V_producer	HH engaged in F+V production (1 = Yes, 0 = No)	0.079	0	1
Income	Total HH monthly income (‘000 Rands)	10.726	0	40
Income_ca	Total HH income per capita (Rands/ca)	4.468	0	40
Main_income_salary	Salaries or wages main income source (1 = Yes, 0 = No)	0.586	0	1
Main_income_grant	Social grants main income source (1 = Yes, 0 = No)	0.200	0	1
Main_income_remit	Remittances main income source (1 = Yes, 0 = No)	0.094	0	1
Main_income_busines	Business main income source (1 = Yes, 0 = No)	0.084	0	1
Main_income_farm	Farming main source of income (1 = Yes, 0 = No)	0.037	0	1
Wealthy	Wealthy status (1 = Reasonably comfortable or wealthy, 0 = Otherwise)	0.249	0	1
Medium	Wealthy status (1 = Neither poor nor rich, 0 = Otherwise)	0.481	0	1
Poor	Wealth status (1 = Poor or very poor, 0 = Otherwise)	0.270	0	1
TLU	Tropical livestock units (TLU)	0.545	0	700
Vehicle	Ownership of a vehicle (1 = Yes, 0 = No)	0.306	0	1
Fridge	Ownership of a refrigerator (1 = Yes, 0 = No)	0.779	0	1
Urban	Geographical location of HH (1 = Urban, 0 = Otherwise)	0.699	0	1
Dist_med_1	Medical facility less than 15 min away (1 = Yes, 0 = No)	0.432	0	1
Dist_med_2	Medical facility at least 15 and less than 30 min away (1 = Yes, 0 = No)	0.417	0	1
Dist_med_3	Medical facility is at least 30 min away from HH (1 = Yes, 0 = No)	0.151	0	1

Source: Authors’ calculations from the 2018 General Household Survey (Stats SA, 2019).

**Table 4:** Determinants of vegetables consumption.

Variables	Freq. of Vegetable Consumption		Chances of Adequate Vegetable Consumption		Frequency of Fruit Consumption		Chances of Adequate Fruit Consumption	
	IRR	Std. Err	Marginal Effect	Std. Err	IRR	Std. Err	Marginal Effect	Std. Err
Mobile_fon_no	1.016 ***	0.004	0.007 **	0.003	1.032 ***	0.006	0.010 ***	0.003
Radio	1.082 ***	0.011	0.038 ***	0.007	1.154 ***	0.019	0.034 ***	0.007
Television	1.041 **	0.018	0.032 ***	0.012	1.217 ***	0.037	0.056 ***	0.012
Internet	1.077 ***	0.022	0.06 1 ***	0.016	1.024	0.029	0.010	0.012
Age	1.002 ***	0.000	0.001 ***	0.000	1.000	0.001	0.000	0.000
Gender	0.990	0.012	−0.001	0.009	0.963 *	0.019	−0.011	0.008
People of colour	0.744 ***	0.015	−0.165 ***	0.015	0.886 ***	0.027	−0.040 ***	0.012
Indian	1.183 ***	0.038	0.160 ***	0.027	1.030	0.050	0.017	0.021
White	1.114 ***	0.024	0.062 ***	0.017	1.071 **	0.033	0.037 ***	0.014
HH_size	0.995	0.004	−0.006 *	0.001	0.936 ***	0.007	−0.018 ***	0.003
Females_15	1.012	0.008	0.012 **	0.005	1.044 ***	0.014	0.009	0.006
Children_5	1.009	0.009	0.003	0.003	1.085 ***	0.017	0.022 ***	0.006
Education	1.001	0.001	0.001	0.006	1.020 ***	0.002	0.006 ***	0.001
Matrics_prop	1.025 ***	0.007	0.008	0.007	1.037 ***	0.011	0.009 **	0.004
Income (logged)	1.036 ***	0.007	0.023 ***	0.005	1.084 ***	0.011	0.025 ***	0.004
Main_income_grant	1.042 **	0.018	0.035 **	0.014	1.104 ***	0.029	0.036 ***	0.011
Main_income_remit	1.036	0.027	0.018	0.019	1.050	0.046	0.015	0.017
Main_income_bus	0.947 ***	0.015	−0.025 **	0.012	0.844 ***	0.024	−0.033 ***	0.011
Main_income_farm	1.023	0.036	0.037	0.027	1.073	0.057	0.030	0.022
Wealthy	1.016	0.013	0.006	0.010	1.130 ***	0.022	0.045 ***	0.008
Poor	0.998	0.013	0.028 ***	0.009	0.963 *	0.022	−0.003	0.009
TLU	1.001 *	0.000	0.000	0.000	1.001	0.000	0.001 *	0.000
Vehicle	1.015	0.014	0.026 **	0.011	1.134 ***	0.025	0.042 ***	0.009
Fridge	1.055 ***	0.016	0.034 ***	0.011	1.166 ***	0.032	0.049 ***	0.011
F+V producer	1.065 ***	0.018	0.067 ***	0.012	0.985	0.029	−0.011	0.012
Urban	0.939 ***	0.012	−0.044 ***	0.009	0.982	0.020	−0.006	0.008
Dist_med_facility_1	1.033 ***	0.011	0.008	0.008	1.061 ***	0.018	0.027 ***	0.007
Dist_med_facility_3	0.877 ***	0.013	−0.072 ***	0.011	0.838 ***	0.022	−0.045 ***	0.010
Constant	1.402 ***	0.081			0.262	0.025		
Observations	17,412		17,412		17,330		17,412	
LR χ^2^ (28)	1362 ***		831 ***		2852 ***		1614 ***	
Pseudo R^2^	0.0195		0.0357		0.0609		0.0815	

*Notes:* ***, **, and * means significant at 1%, 5%, and 10% levels, respectively; IRR means Incidence-Rate Ratio. Source: Authors’ calculations from the 2018 General Household Survey (Stats SA, 2019).
